# Alteration of the copy number and deletion of mitochondrial DNA in human hepatocellular carcinoma

**DOI:** 10.1038/sj.bjc.6601838

**Published:** 2004-05-04

**Authors:** P H Yin, H C Lee, G Y Chau, Y T Wu, S H Li, W Y Lui, Y H Wei, T Y Liu, C W Chi

**Affiliations:** 1 Institute of Pharmacology, National Yang-Ming University, Taiwan, Republic of China; 2 Department of Surgery, National Yang-Ming University, Taiwan, Republic of China; 3 Institute of Biochemistry, Chung Shan Medical University, Taiwan, Republic of China; 4 Department of Biochemistry and Center for Cellular and Molecular Biology, National Yang-Ming University, Taiwan, Republic of China; 5 Department of Surgery, Taipei Veterans General Hospital, Taiwan, Republic of China; 6 Department of Medical Research and Education, Taipei Veterans General Hospital, Taipei 11217, Taiwan, Republic of China

**Keywords:** hepatoma, gender, mitochondrial biogenesis, somatic mutation, alcohol

## Abstract

Somatic mutations in mitochondrial DNA (mtDNA) have been detected in hepatocellular carcinoma (HCC). However, it remains unclear whether mtDNA copy number and mitochondrial biogenesis are altered in HCC. In this study, we found that mtDNA copy number and the content of mitochondrial respiratory proteins were reduced in HCCs as compared with the corresponding non-tumorous livers. MtDNA copy number was significantly reduced in female HCC but not in male HCC. Expression of the peroxisome proliferator-activated receptor *γ* coactivator-1 was significantly repressed in HCCs (*P*<0.005), while the expression of the mitochondrial single-strand DNA-binding protein was upregulated, indicating that the regulation of mitochondria biogenesis is disturbed in HCC. Moreover, 22% of HCCs carried a somatic mutation in the mtDNA D-loop region. The non-tumorous liver of the HCC patients with a long-term alcohol-drinking history contained reduced mtDNA copy number (*P*<0.05) and higher level of the 4977 bp-deleted mtDNA (*P*<0.05) as compared with non-alcohol patients. Our results suggest that reduced mtDNA copy number, impaired mitochondrial biogenesis and somatic mutations in mtDNA are important events during carcinogenesis of HCC, and the differential alterations in mtDNA of male and female HCC may contribute to the differences in the clinical manifestation between female and male HCC patients.

Hepatocellular carcinoma (HCC) is one of the most common cancers in Taiwan. The incidence of HCC in males is 3–6 times higher than that in females. Lower tumour recurrence rate and better 5-year survival after resection of HCC were found for female patients as compared to males (Nagasue *et al*, 1989; Jwo *et al*, 1992). However, the molecular mechanism underlying these observations is not clear.

Recently, somatic mutations in the mitochondrial DNA (mtDNA) have been identified in various human cancers such as colon cancer, gastric cancer and HCC (Polyak *et al*, 1998; Fliss *et al*, 2000; Nishikawa *et al*, 2001; Sanchez-Cespedes *et al*, 2001). Human mtDNA is a 16.5-kb circular double-stranded DNA molecule, which contains genes coding for 13 polypeptides involved in respiration and oxidative phosphorylation, two rRNAs and a set of 22 tRNAs that are essential for protein synthesis in mitochondria (Anderson *et al*, 1981). There are 2–10 copies of mtDNA in each mitochondrion, with up to several thousand mitochondria per human cell. Moreover, nuclear DNA provides all of the gene products necessary for mtDNA replication and transcription, as well as other components of the oxidative phosphorylation system (Scarpulla, 1997). These nuclear DNA products include the mitochondrial single-stranded DNA-binding protein (mtSSB), mitochondrial transcription factor A (mtTFA), nuclear respiratory factor-1 (NRF-1) and peroxisome proliferator-activated receptor *γ* coactivator-1 (PGC-1). However, alterations in the mtDNA content and in the factors involved in the stimulation of the mtDNA replication and transcription within HCC and the corresponding non-tumorous liver remain obscure.

To evaluate any alteration in the quality or quantity of mtDNA within HCC and the corresponding non-tumorous liver between female and male patients, we determined the copy number and mutations of mtDNA in the tumour tissues and in the corresponding non-tumorous liver tissues from both male and female HCC patients. Alterations in gene expression of the nuclear DNA-encoded factors involved in the regulation of mtDNA replication and transcription were also analysed.

## MATERIALS AND METHODS

### Human HCC tissues

Histologically confirmed HCC and corresponding non-tumorous liver tissues were obtained from 18 patients, including nine males and nine females. All the tissues were kept in liquid nitrogen immediately after surgical resection at the Taipei Veterans General Hospital, according to a protocol approved by the committee for conducting human research at the hospital.

### Determination of MtDNA copy number

Total DNA was extracted from tissues using the TRIzol Reagent (Life Technologies) according to the instructions of the manufacturer. The mtDNA copy number was determined using a competitive polymerase chain reaction (PCR) method (Lee *et al*, 1998, 2004). In brief, a known amount of the internal DNA standard was introduced with the DNA sample into the PCR reaction mixture. The PCR reactions were carried out for 25 cycles in a 50 *μ*l reaction mixture containing 200 ng DNA, 200 *μ*M of each dNTP and 40 pmol of each primer (BA1, L495, ND1L and ND1 H) (Table 1

**Table 1:** Sequences of the oligonucleotide primers used in this study

**Target gene**	**Primer name**	**Sequences (5′–3′)**
*β*-actin	BA1	CATGTGCAAGGCCGGCTTCG
	L495	CTGGGTCATCTTCTCGCGGT
MtDNA (ND1)	ND1L	TCTCACCATCGCTCTTCTAC
	ND1H	TTGGTCTCTGCTAGTGTGGA
MtDNA (D-loop)	L16190	CCCCATGCTTACAAGCAAGT
	L76	CACGCGATAGCATTGCGAGACGCTG
	H602	GCTTTGAGGAGGTAAGCTAC
MtDNA (Deletion)	L8150	CCGGGGGTATACTACGGTCA
	H13650	GGGGAAGCGAGGTTGACCTG
MtDNA (ND1)	L3304	AACATACCCATGGCCAACCT
	H3836	GGCAGG AGTAATCAGAGGTG
*β*-actin	BAF	TGGCATTGCCGACAGGAT
	BAR	GCTCAGGAGGAGCAATGATCT
mtSSB	mtSSBF	CCTCAGAGACGTGGCATATCAA
	mtSSBR	CGCCTCACATTATTTTTATCCATGT
NRF-1	NRF-1F	TTGGAGAATGTGGTGCGTAAGT
	NRF-1R	GAGAGGCGGCAGTTCTGAGT
mtTFA	mtTFAF	CCAAAAAGACCTCGTTCAGCTTA
	mtTFAR	CTTTACAGTCTTCAGCTTTTCCTGC
PGC-1	PGC-1F	CCAAATGACCCCAAGGGTTC
	PGC-1R	TATGAGGAGGAGTGGTGGGTG

), 1.0 U of *Taq* DNA polymerase, 50 mM KCl, 1.5 mM MgCl_2_, 10 mM Tris–HCl (pH 9.0), 0.1% Triton X-100 and 0.01% (w v^−1^) gelatin. The intensities of the PCR products of the target and internal standard DNAs were analysed. The intensity ratio of the DNA bands was used to calculate the mtDNA copy number.

### Quantitative real-time reverse transcription (RT)–PCR analysis

Total RNA was extracted from tissues using the TRIzol Reagent (Life Technologies), according to the instructions of the manufacturer. The first-strand cDNA was synthesised with a random primer pd(N)_6_ in a 33 *μ*l mixture using RT–beads (Amersham Pharmacia Biotech). Real-time quantitative PCR was performed using the ABI PRISM 7700 (PE Applied Biosystems) and results were analysed with the accompanying software. The primers for *β*-actin, PGC-1, NRF-1, mtTFA and mtSSB are listed in Table 1. The DNA-intercalating SyBr green reagent was used for detection of the RT–PCR product. The PCR cycle used was as follows: 50°C (2 min), 95°C (10 min), followed by 40 cycles of 95°C (15 s), 60°C (1 min). The alteration in gene expression was obtained using the ΔΔCt method in which all samples are first normalised to the level of *β*-actin in each sample. Relative normalised units were then compared between the tumour tissue and the non-tumorous liver tissue obtained from the same HCC patient.

### Immunoblot analysis

Total protein concentrations of tissue lysates were determined with the Bradford reagent (Bio-Rad). In all, 10 *μ*g of each lysate were subjected to SDS–PAGE/immunoblot analysis using mouse monoclonal antibodies specific for the 72 kDa subunit of the succinate-ubiquinol oxidoreductase (complex II, A-11142, Molecular Probes) and the core 2 subunit of the ubiquinol-cytochrome *c* oxidoreductase (complex III, A-11143, Molecular Probes), respectively, and the secondary horseradish peroxidase-conjugated sheep-anti-mouse antibody (Amersham Biosciences) and an enhanced chemiluminescence detection method (ECL Western Blotting System, Amersham Biosciences).

### Direct sequencing analysis of MtDNA D-loop region

The primers L16190 and H602 were used for the amplification of a 982 bp DNA fragment from the D-loop region of mtDNA. All PCR products were purified and sequenced directly with the AmpliCycle sequencing kit (Perkin-Elmer) according to the instructions of the manufacturer. Both strands of the sequence were, respectively, analysed by using the primers L16190 and H602, and the primer L76 was used for confirming the alteration in the poly C tract.

### Detection and quantification of the 4977 bp-deleted MtDNA

Using a previously described PCR method (Lee *et al*, 2001), the primers L8150 and H13650 were used for the amplification of a 524 bp PCR product from the 4977 bp-deleted mtDNA. The primers L3304 and H3836 were used for the amplification of a 533 bp DNA fragment from total mtDNA. The proportion of the 4977 bp-deleted mtDNA was determined by the ratio of the highest dilution fold that allowed the 524 bp PCR product amplified from the 4977 bp-deleted mtDNA to be visible on the gel to that which allowed the 533 bp PCR product to be visibly amplified from the total mtDNA under identical conditions.

### Statistical analysis

Data are presented as mean±s.e.m. except where indicated. Comparisons among multiple groups were made by an analysis of Student's *t*-test. Stepwise multiple regression analysis was adopted to identify the variable with statistical significance. The Scientific Package for Social Sciences (SPSS), version 10.0, software for Windows was used. A value of *P*<0.05 was considered statistically significant.

## RESULTS

### Alteration in mtDNA copy number of HCC

**Figure 1:**
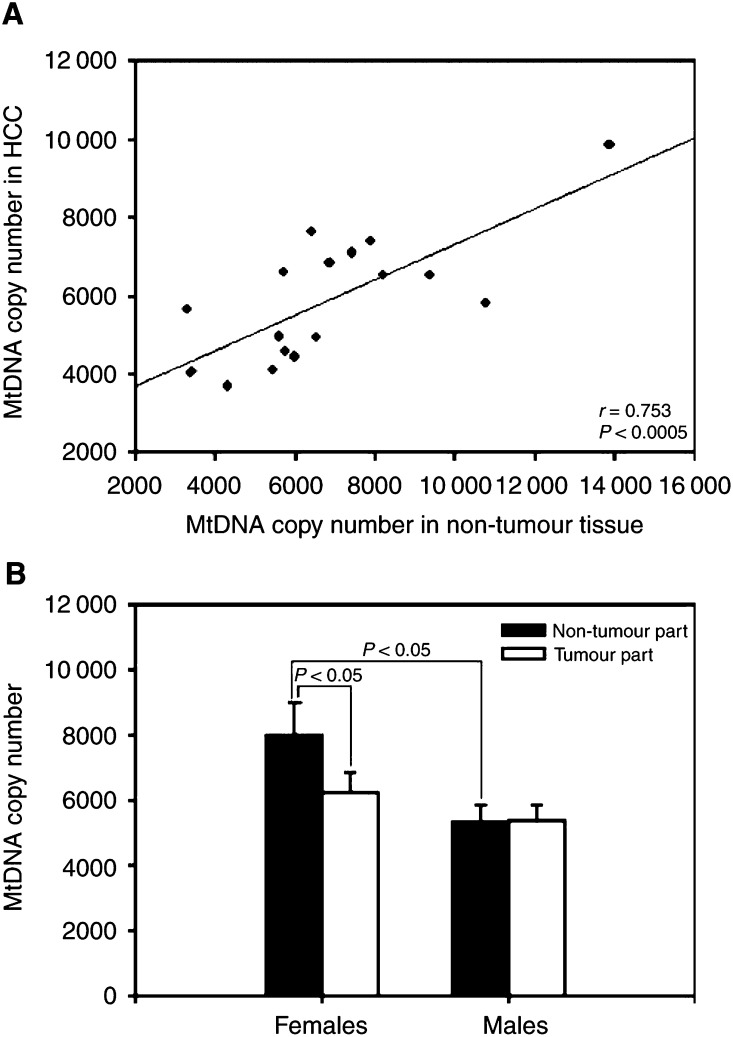
MtDNA copy number in HCC. Panel **A** shows a positive correlation between the copy number of mtDNA within the tumour tissues and the corresponding non-tumorous tissues (*r*=0.753; *P*<0.0005). Panel **B** shows the mean copy number of mtDNA within the tumour tissues and the corresponding non-tumorous tissues from the female and male HCC patients. The results are presented as mean±s.e.m. Comparisons between the group means were analysed by Student's *t*-test.

To evaluate whether any changes in the abundance of mtDNA occurred within the tumour tissues of HCC patients, we analysed the mtDNA copy number of the tumour tissues and the corresponding non-tumorous liver tissues by a competitive PCR technique. We found a direct correlation between the copy number of mtDNA from the tumour and the corresponding non-tumorous liver tissues of these HCC patients (*r*=0.753, *P*<0.0005, Figure 1A

). However, the mean copy number of mtDNA in the non-tumorous tissues of the male HCC patients (5308±484) was significantly lower than that of the female patients (8027±969) (*P*<0.05, Figure 1B). Moreover, the mean copy number of mtDNA in the tumour tissues (6262±614) of the female HCC patients was significantly lower than that of the corresponding non-tumorous liver tissues (*P*<0.05), though no significant difference in the mtDNA copy number was found between the tumour and the corresponding non-tumorous liver tissues in the male HCC patients (Figure 1B).

### Respiratory protein contents were reduced in HCC

**Figure 2:**
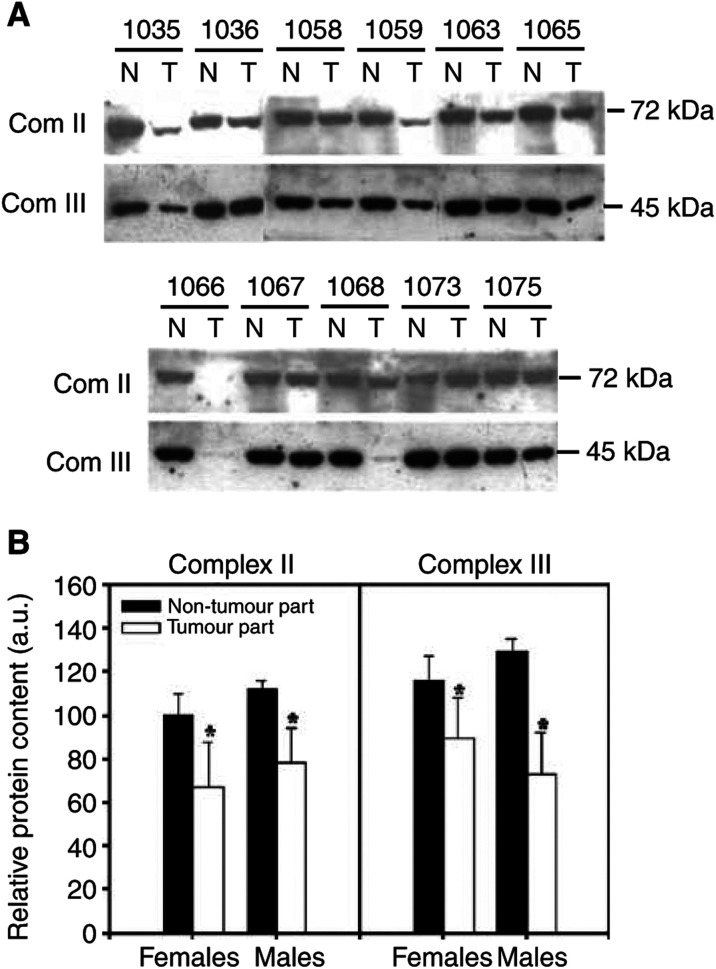
Mitochondrial respiratory proteins in HCC. Panel **A** shows the relative content of the mitochondrial respiratory proteins, the 72 kDa subunit of the succinate-ubiquinol oxidoreductase (complex II) and the core 2 subunit of the ubiquinol-cytochrome *c* oxidoreductase (complex III), determined by immunoblot analysis described in Materials and methods. Panel **B** shows the histogram of the protein content (expressed as arbitrary units, a.u.) of each mitochondrial respiratory protein in non-tumorous and tumour tissues from five female and six male HCC patients. The results are presented as mean±s.e.m. ^*^ represents a significant decrease in the protein content of the tumour tissues as compared with that of the corresponding non-tumorous tissues (*P*<0.05, Student's *t*-test).

Immunoblot analyses indicated that the cellular content of the nuclear-encoded mitochondrial respiratory proteins, the 72 kDa subunit of the succinate-ubiquinol oxidoreductase (complex II) and the core 2 subunit of the ubiquinol-cytochrome *c* oxidoreductase (complex III) were significantly reduced in HCCs (*P*<0.005 and *P*<0.004, respectively) when compared with the corresponding non-tumorous livers (Figure 2A

). Further analyses revealed that decreased expression of the mitochondrial respiratory proteins in tumour tissues was found in both male and female HCC patients as compared with those in the corresponding non-tumorous livers (Figure 2B). The concomitant reduction in the copy number of mtDNA and the content of mitochondrial respiratory proteins (Figures 1 and 2) strongly suggests that the biogenesis of mitochondria was repressed in human HCC.

### MtSSB expression was not correlated with mtDNA content in HCC

**Figure 3:**
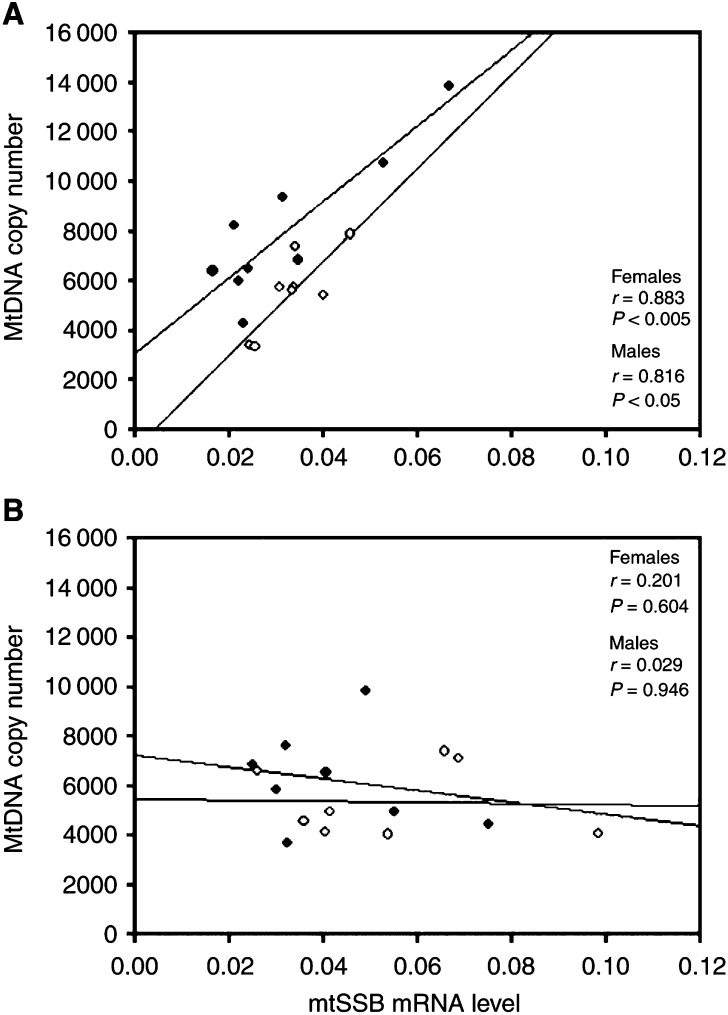
Correlation between the expression of mtSSB mRNA and mtDNA copy number. Panel **A** shows a positive correlation between the expression of mtSSB mRNA and the copy number of mtDNA within the non-tumorous tissues of the female HCC patients (the closed symbols, *r*=0.883; *P*<0.005) and of the male HCC patients (the open symbols, *r*=0.816; *P*<0.05), respectively. Panel **B** shows a loss of the relationship between the expression of mtSSB mRNA and the copy number of mtDNA within the tumour tissues of the female HCC patients (the closed symbols, *r*=0.201; *P*=0.604) and of the male HCC patients (the open symbols, *r*=0.029; *P*=0.946), respectively.

Mitochondria rely upon nuclear genes to regulate their biogenesis and maintenance of mtDNA. Using a real-time quantitative RT–PCR method, we found that the expression of mtSSB mRNA correlated directly with the copy number of mtDNA in the non-tumorous tissues of the female HCC patients (*r*=0.883, *P*<0.005) and the male HCC patients (*r*=0.816, *P*<0.05), respectively (Figure 3A

). In contrast, no correlation was found in the expression of mtSSB mRNA and the abundance of mtDNA in either the male or female HCC tumour tissues (Figure 3B).

### PGC-1 expression was repressed in HCC

**Figure 4:**
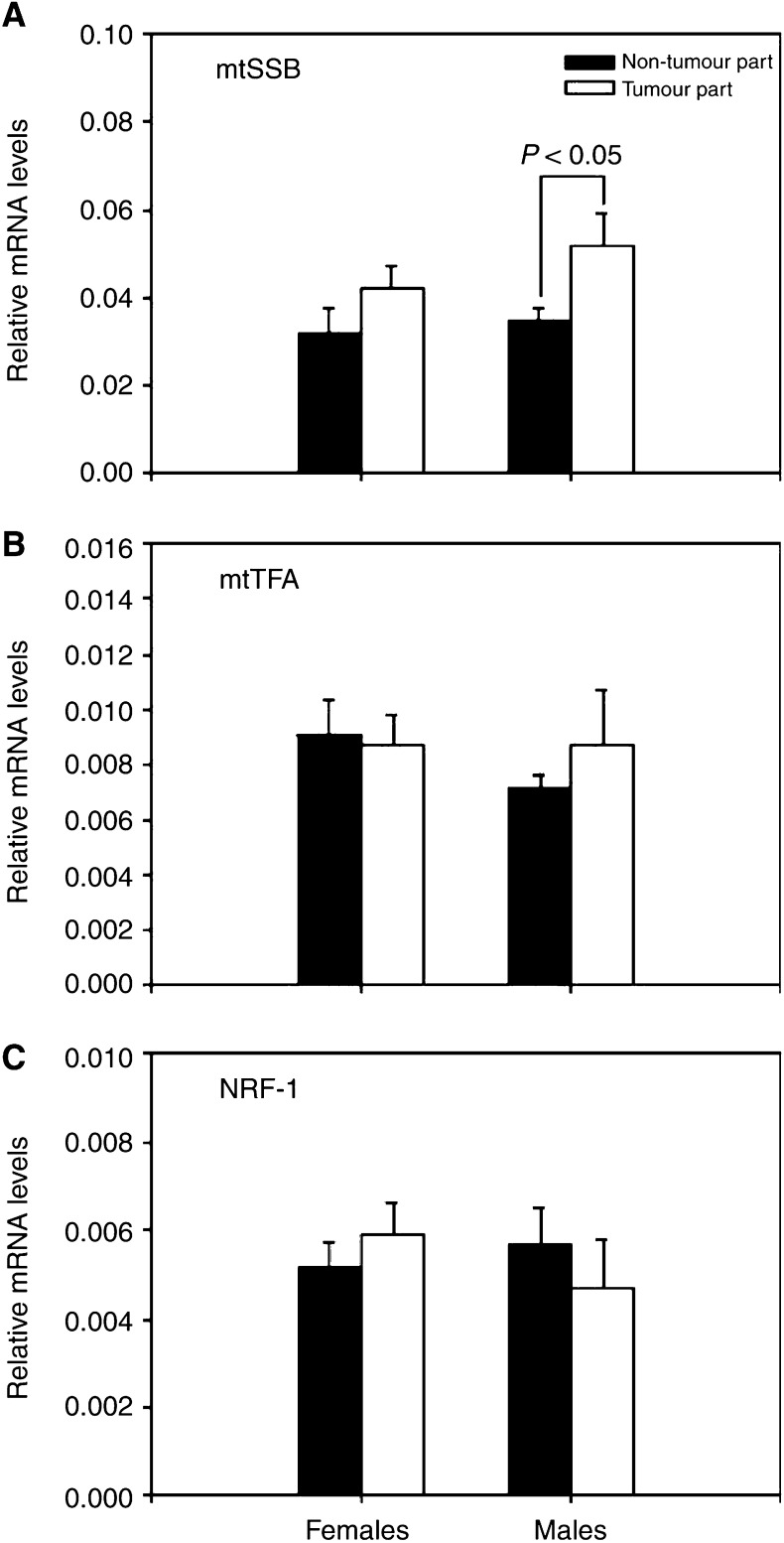
Alterations in the mRNA expressions of mtSSB, mtTFA and NRF-1 in HCC. Alterations in the mRNA expressions of mtSSB (panel **A**), mtTFA (panel **B**) and NRF-1 (panel **C**) were analysed by the ΔΔCt method, as described in Materials and methods. Data are first normalised to the level of *β*-actin in each sample. The results are presented as mean±s.e.m. Comparisons between the group means were analysed by Student's *t*-test. A value of *P*<0.05 was considered statistically significant.

**Figure 5:**
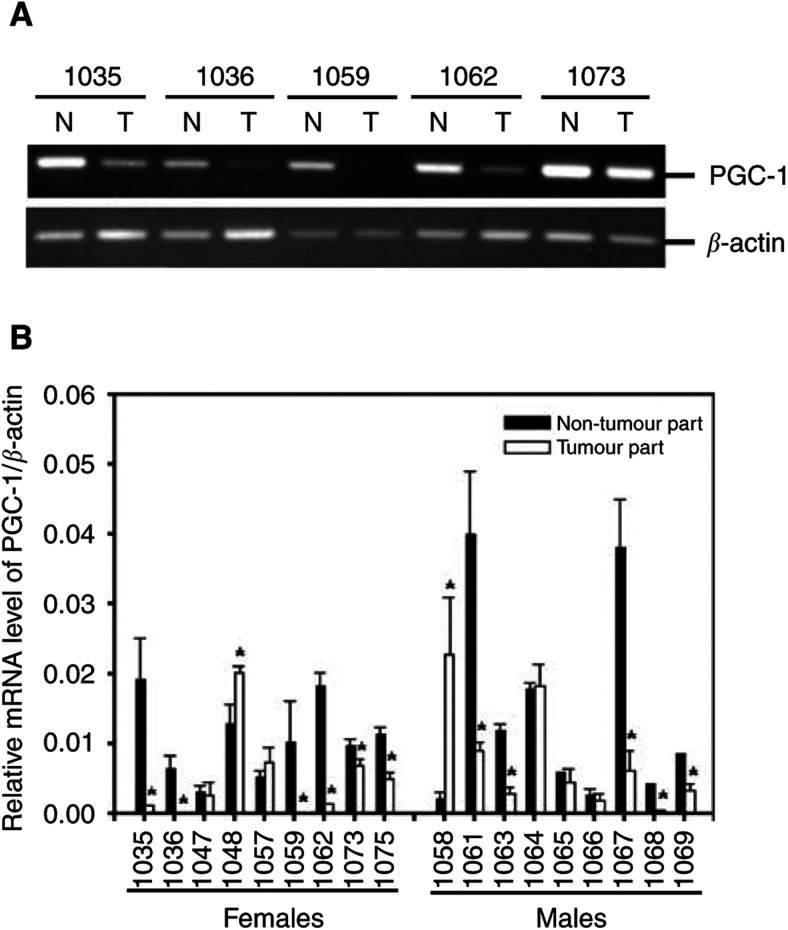
Alteration in the mRNA expression of PGC-1 in HCC. Alteration in the mRNA expression of PGC-1 was analysed by RT–PCR method (panel **A**) and was quantified by the ΔΔCt method (panel **B**) as described in Materials and methods. Data are first normalised to the level of *β*-actin in each sample. The results are presented as mean±s.e.m. ^*^ represents a significant alteration in the mRNA level of PGC-1 of the tumour tissues as compared with that of the corresponding non-tumorous tissues (*P*<0.05, Student's *t*-test).

Further analysis showed that the average mRNA expression of mtSSB was significantly upregulated in the tumour tissues of the male HCC patients as compared with that in the corresponding non-tumorous tissues (*P*<0.05), but the average mRNA levels of mtSSB were only slightly increased in the tumour tissues of the female HCC patients (Figure 4A

). Moreover, analyses of the mRNA expressions of the NRF-1 and mtTFA genes in the tumour and the corresponding non-tumorous liver tissues of the HCC patients showed that no significant difference was observed (Figure 4B and C). In contrast, a significant reduction in the mRNA level of the PGC-1 of the tumour tissues was observed in 61% (11 out of 18) of the HCC patients, including six females and five males (Figure 5A and B

).

### Somatic alterations in the D-loop of mtDNA in HCC

To analyse the qualitative alteration of mtDNA in the tumour tissues of HCC patients, we used a direct sequencing method to determine the somatic mutation and sequence variation in the D-loop region (from nucleotide position (np) 16 190 to np 602) of mtDNA in the tumour and corresponding non-tumorous liver tissues of the HCC patients. We detected a somatic deletion of a cytidine in a poly C tract starting at np 303 in 22% (four out of 18) of HCC tumour tissues. Microsatellite DNA alterations in the D-loop region were found in 11% (one out of nine) of tumour tissues of the female patients and in 33% (three out of nine) of tumour tissues of the males. All the nucleotide changes detected in the present study were homoplasmic.

### Alteration in accumulation of mtDNA 4977 bp deletion in HCC

**Figure 6:**
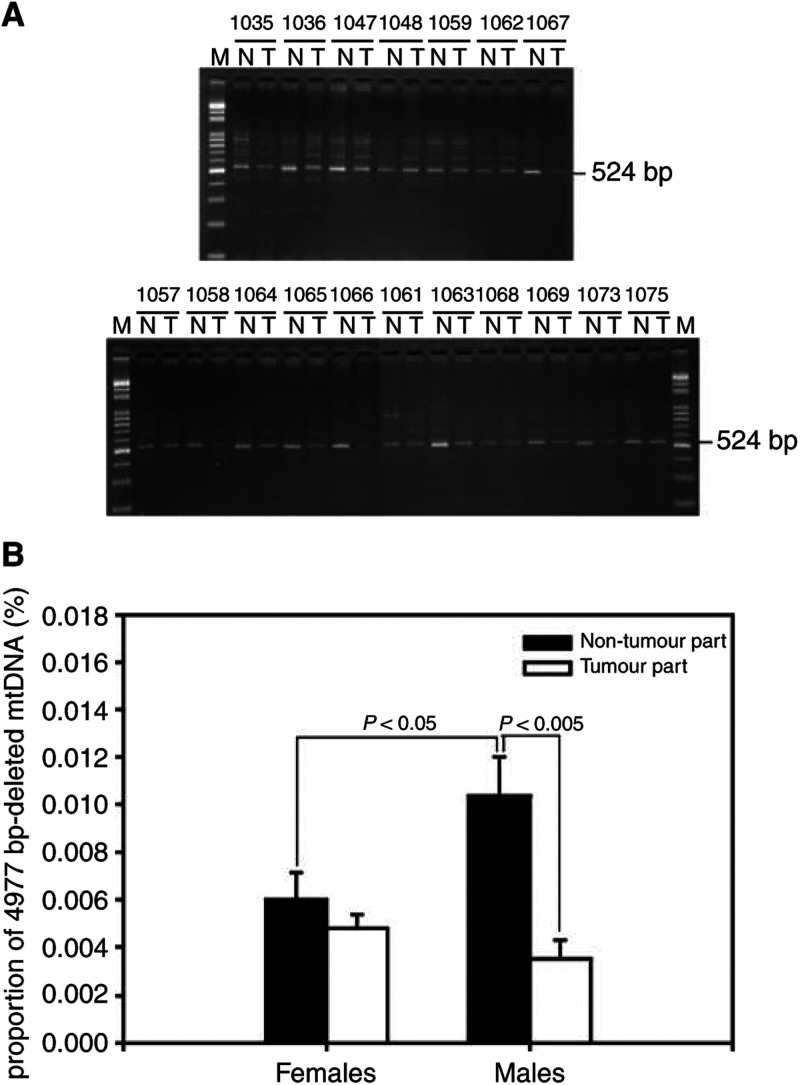
MtDNA 4977 bp deletion in HCC MtDNA with the 4977 bp deletion was detected by the PCR technique described in Materials and methods. Using the primers L8150 and H13650, a 524 bp PCR product amplified from the 4977 bp-deleted mtDNA in the tumour part (T) and in the corresponding non-tumorous tissue (N) were separated on a 1.5% agarose gel and detected under UV transillumination after ethidium bromide staining. M: 100 bp DNA ladder. The numbers above the gel lanes indicate the patients (panel **A**). The mean proportions of the 4977 bp-deleted mtDNA in the tumour tissues were compared with the corresponding non-tumour tissues (panel **B**). The results are presented as mean±s.e.m. Comparisons between the group means were analysed by Student's *t*-test.

We further observed that the common 4977 bp deletion occurred and accumulated in the tumour tissues and in the corresponding non-tumorous liver tissues of HCC patients. In the 36 liver samples examined, the 4977 bp-deleted mtDNA was detected in all of the tumours and the non-tumorous liver tissues of the HCC patients (Figure 6A

). Interestingly, the mean proportion of the 4977 bp-deleted mtDNA in the non-tumorous tissues of the male HCC patients (0.0104±0.0016%) was significantly higher than that of the female patients (0.0060±0.0011%) (*P*<0.05, Figure 6B). Moreover, the mean proportion of the 4977 bp-deleted mtDNA was significantly decreased in the tumour tissues (0.0035±0.0008%) as compared with that in the corresponding non-tumorous liver tissues (0.0104±0.0016%) of the male HCC patients (*P*<0.005), but the phenomenon was not significant in the female HCC patients (Figure 6B).

**Figure 7:**
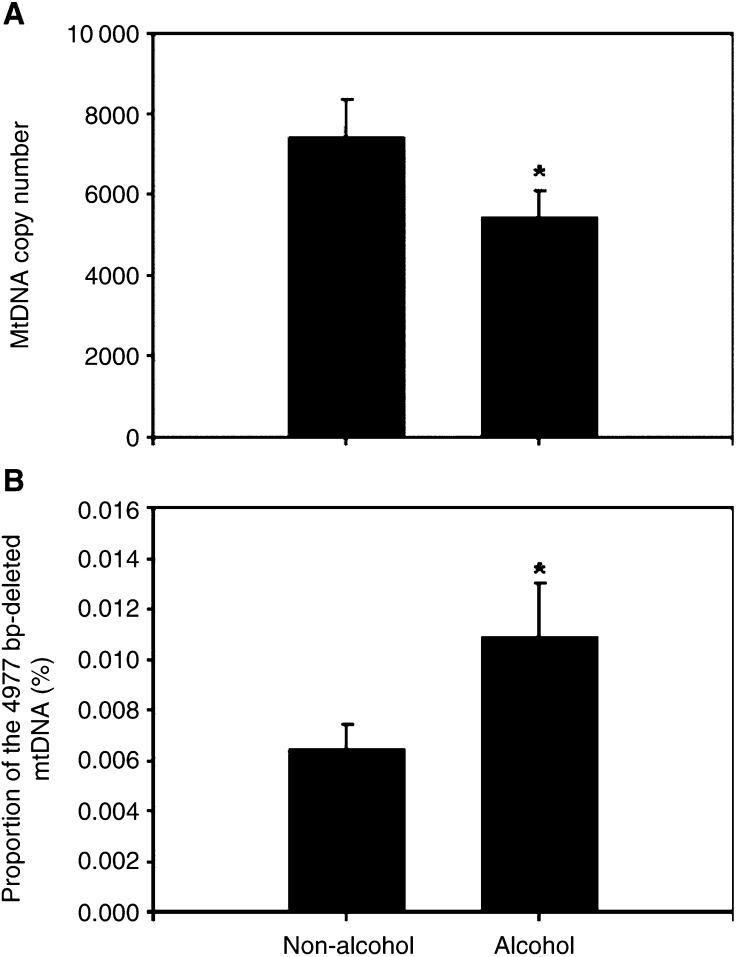
The effect of alcohol consumption on the level of mtDNA copy number and the accumulation of the 4977 bp-deleted mtDNA in the liver tissues. The level of mtDNA copy number (**A**) and the proportion of the 4977 bp-deleted mtDNA (**B**) were, respectively, determined by the PCR techniques described in Materials and methods. The mean copy number of mtDNA in the non-tumorous tissues of seven HCC patients with a history of long-term drinking of alcohol (alcohol group) was significantly lower than that of 11 HCC patients without a history of long-term drinking of alcohol (non-alcohol group) (^*^P<0.05, Student's *t*-test). In contrast, the mean proportion of the 4977 bp-deleted mtDNA in the non-tumorous tissues of the alcohol group was significantly higher than that of the non-alcohol group (^*^P<0.05, Student's *t*-test).

In the present study, 39% (seven out of 18) of the HCC patients have a history of long-term drinking of alcohol. Analyses of the relation of alcohol consumption with the level of mtDNA copy number and the accumulation of the mtDNA with the 4977 bp deletion revealed that the mean copy number of mtDNA in the non-tumorous tissues of seven HCC patients with a history of long-term drinking of alcohol (alcohol group, 5447±632) was significantly lower than that of 11 HCC patients without a history of long-term drinking of alcohol (non-alcohol group, 7444±904) (*P*<0.05, Figure 7A

). In contrast, the mean proportion of the 4977 bp-deleted mtDNA in the non-tumorous tissues of the alcohol group (0.0109±0.0021%) was significantly higher than that of the non-alcohol group (0.0065±0.0010%) (*P*<0.05, Figure 7B).

## DISCUSSION

Mitochondria are involved in multiple cellular processes such as energy metabolism, apoptosis and generation of reactive oxygen species (ROS). Alterations in oxidative phosphorylation of tumour cells are proposed to play a causative role in tumour formation or in the manifestation of clinical phenotype and malignant potential of the tumour (Augenlicht and Heerdt, 2001). Our results demonstrated for the first time that the copy number of mtDNA, the content of mitochondrial respiratory proteins and mRNA expression of PGC-1 are significantly decreased in HCC. It was recently observed that there are differential expressions of mitochondrial proteins in carcinomas (Cuezva *et al*, 2002); it is possible that the expression of mitochondrial citrate synthase, a standard usually used in mitochondrial disease studies, is changed in the mitochondria of carcinomas. Consequently, we examined the changes in the content of mitochondrial respiratory proteins in relation to total cellular protein rather than to the citrate synthase. We found that the cellular content of mitochondrial respiratory complex II and complex III subunits was significantly reduced in HCC as compared with the corresponding non-tumorous livers. In addition, it has been demonstrated that PGC-1 is required for mitochondrial biogenesis (Wu *et al*, 1999) and for the regulation of hepatic gluconeogenesis (Yoon *et al*, 2001). These findings suggest that mitochondrial biogenesis is impaired during liver carcinogenesis. Our results not only support the observation (Warburg, 1956) that cancer cells have a respiratory deficiency, but also agree with the observation of Cuezva *et al* (2002), who reported that the expression level of the mitochondrial *β*-F1-ATPase and Hsp 60, and the cellular content of mtDNA were reduced in HCCs.

In the analysis for nucleotide changes located in the D-loop region of mtDNA, 1-bp deletion of a mononucleotide repeat (poly C) tract at np 303–309 in the D-loop region of mtDNA was detected in four out of 18 (22%) HCC patients. The poly C tract was recently reported to harbour deletions or insertions at a very high frequency in several primary tumours (Sanchez-Cespedes *et al*, 2001). Moreover, this region was found to be highly susceptible to oxidative damage as compared with the other regions of mtDNA (Mambo *et al*, 2003). These results, together with the observation that oxidative stress was high in HCC (Toyokuni *et al*, 1995), suggest that oxidative damage may contribute to the changes of the mononucleotide repeat at np 303–309 in the D-loop region of mtDNA in HCCs.

MtSSB is necessary for mtDNA replication and the expression of mtSSB is strictly regulated and correlates directly with mtDNA content (Schultz *et al*, 1998). Our results showed that the expression of mtSSB correlated with the mtDNA copy number in the non-tumorous tissue, but not in the tumour tissue of HCC patients (Figure 3). In contrast, the expressions of mtTFA and its upstream transcription factor NRF-1 were not significantly altered in HCC (Figure 3). MtTFA is required for efficient heavy- and light-strand transcription and also participates in the replication and maintenance of mtDNA (Virbasius and Scarpulla, 1994). NRF-1 has been demonstrated to be an important activator of promoters for mtTFA and components of the mitochondrial electron transport chain (Scarpulla, 1997). The findings indicate that a loss in the strict regulation of the mtSSB expression may contribute to a decreased copy number of mtDNA in HCC.

In this study, the proportion of mtDNA with the 4977 bp deletion was significantly reduced in the tumour tissues of the male HCC patients as compared with the corresponding non-tumorous tissues. Similarly, a slight decrease in the proportion of deleted mtDNA was also observed in the tumour tissues of the female HCC patients. Our results are in agreement with previous findings in HCC (Kotake *et al*, 1999) and in human oral cancers (Lee *et al*, 2001). Moreover, it was found that the proportion of mtDNA with deletion in the more rapidly dividing tissues was lower than that of tissues with a slow turnover rate (Kotake *et al*, 1999). Thus, the decrease in the proportion of mtDNA with deletion in tumour tissues may have resulted from that the deleted mtDNA molecules were diluted in the HCC cells after high mitotic segregation.

Heavy alcohol consumption has been documented as one of HCC's risk factors (Chen *et al*, 1997). We found that the HCC patients with a history of long-term drinking of alcohol have a significantly decreased mtDNA copy number and an obviously increased level of mtDNA deletion in their liver (Figure 7A and B). It has been demonstrated that acute or chronic ethanol intoxication causes hepatic oxidative stress and mitochondrial dysfunction in human and experimental animals (Cederbaum, 1999). Ethanol induced hepatic mtDNA depletion after a single binge in mice (Demeilliers *et al*, 2002), or chronic ethanol intoxication in aged rats (Cederbaum, 1999). Multiple hepatic mtDNA deletions were frequently observed in the liver tissue and white blood cells (WBC) obtained from patients with alcoholic liver disease (ALD) (Mansouri *et al*, 1997). Together, these results suggest that ethanol intoxication might induce a decrease in the copy number of mtDNA and an increase in the proportion of mtDNA deletion in the liver tissues of male HCC patients.

Several studies have provided evidence that mtDNA plays an important role in cellular sensitivity to cancer therapies (Singh *et al*, 1999). It has been suggested that the mitochondrion could be the integrator of many signals that have a potential impact on tumour-related process. Our results suggest that alterations in the mutations and copy number of mtDNA in the tumour tissues might influence tumour formation, phenotype and the sensitivity to chemotherapeutic agents.

In summary, our results demonstrated that reduced mtDNA copy number, impaired mitochondrial biogenesis and somatic mutations in mtDNA occurred concomitantly in HCC tissues. Moreover, a decreased mtDNA content combined with somatic mutations in the D-loop region of mtDNA of the non-tumorous livers or HCC cells might contribute to the difference in the clinical manifestation, progression and mortality rate between female and male HCC patients.
